# Sexual dysfunction in men with type II diabetes

**DOI:** 10.22088/cjim.11.3.295

**Published:** 2020-05

**Authors:** Adele Bahar, Forouzan Elyasi, Mahmood Moosazadeh, Ghasem Afradi, Zahra Kashi

**Affiliations:** 1 Diabetes Research Center, Mazandaran University of Medical Sciences, Sari, Iran; 2 Sexual and Reproductive Health Research Center, Addiction Institute, School of Medicine, Mazandaran University of Medical Sciences, Sari, Iran; 3 Health Sciences Research Center, Addiction Institute, Mazandaran University of Medical Sciences, Sari, Iran; 4 Department of Internal Medicine, School Of Medicine, Mazandaran University of Medical Sciences, Sari, Iran

**Keywords:** Diabetes, Sexual dysfunction, erectile dysfunction, Prevalence

## Abstract

**Background:**

Diabetes mellitus (DM) is a chronic disease inducing short-term and long-term complications including sexual dysfunction (SD) which can consequently reduce patients’ quality of life. Given the limited literature on frequency of SD in men experiencing diabetes in northern Iran, the present study was conducted in the city of Sari in Mazandaran Province, with the aim of investigating SD in men with type II diabetes.

**Methods:**

Using a descriptive cross-sectional research design, a total number of 350 male patients suffering from type II diabetes referring to endocrinology clinics in the city of Sari in. The patients were requested to fill out the demographic questionnaire, depression, anxiety and stress scale-21 items (DASS-21) and the 15-question International Index of Erectile Function (IIEF). The data were analyzed using the IBM SPSS statistics software

**Results:**

The average period of time in which the patients were facing diabetes was 3.65±5.75 years. The IIEF mean score was equal to 16.98±43.79. Erectile dysfunction (ED) was also evident in 152 patients (62.2%). Moreover, increase in age had significantly decreased the IIEF scores (p<0.001). The chance of being affected with ED among diabetic patients above 50 was 11.21 times as much as those below 50 years of age (odds ratio (OR): 11.21, 95% confidence interval (CI): 6.40-19.62).

**Conclusion:**

Concerning the high prevalence rate of ED in men suffering from type II diabetes, doctors are required to directly ask them about sexual disorders in follow-up visits. Furthermore, using screening questionnaires can be helpful in identifying this problem.

Diabetes mellitus (DM) is considered as one of the most common chronic diseases as well as endocrine and metabolic disorders threatening global health (1). Today, diabetes is the fifth cause of mortality in Western societies and also the fourth common cause of visiting a doctor. This condition also brings about several complications due to hyperglycemia in a way that approximately 4 million annual deaths occur because of diabetes complications, which makes up about 9% of mortality rates all around the world. Such complications include cardiovascular diseases (CVDs), heart attacks, strokes, kidney failure, non-traumatic lower extremity amputations, retinopathy, nephropathy, neuropathy, blindness, reduction of life expectancy, and sexual impotence (2, 3). Accordingly, men with diabetes are also vulnerable to a wide variety of sexual problems (4). Erectile dysfunction (ED), reduced sexual desire, orgasmic disorder, and retrograde ejaculation are among the complications of variable incidence of such problems in men suffering from diabetes (5, 6). In this respect, ED is described as a persistent inability (more than 6 months) to attain and maintain an erection sufficient to have satisfactory sexual function (7). Erectile disorder may be also a sign of diabetes and may further predict subsequent neural consequences (5, 6). Pathophysiology of ED in diabetes is multifactorial and contains vascular, hormonal, and neural complications. 

Diabetic neuropathy can similarly cause autonomic and somatic neural disorders which are of importance for erection. Besides diabetes can bring about disorders in relaxation of cavernous smooth muscles as a result of the nitric acid produced from endothelium, which may be a side effect of glycated products (8, 13). 

Recent evidence indicates that men with diabetes may be in growing danger of reduction of testosterone levels (hypogonadism) in addition to problems related to arteries and nerves supporting the penis (14, 15). Although an exact mechanism of this effect has not been completely identified, hypogonadism in such men may indirectly mitigate levels of pituitary hormones, responsible for stimulating testosterone production in testicles (16). 

Low levels of testosterone may also lead to loss of sex drive or cause ED either directly or indirectly (5, 17). However, the prevalence rates of sex drive, orgasmic disorders, and ejaculation problems have not been exactly determined (18). ED occurs in a considerable number of diabetic men, and its incidence estimation is very high in different studies, ranging from 20 to 71%. ED significantly affects quality of life (QoL) in men with diabetes (18). 

The incidence of ED in men suffering from diabetes is almost three times as much as the general population (19, 20). For example, with the aim of determining levels of sexual dysfunction (SD) in male patients affected with type II diabetes, in 2014, Fallahi et al. carried out a descriptive study on 69 male patients in the city of Yazd, Iran, using questionnaires containing patients’ demographic characteristics information and the International Index of Erectile Function (IIEF). 

According to their study, most of the participants had trouble with erection as well as the number of times they had experienced sexual intercourse (21). In Mazandaran Province, Iran, Elyasi et al. (2015) also investigated SD in women suffering from type II diabetes and reported that 78.7% of the women had SD (22). 

Concerning the high prevalence rate of SD in diabetic patients, given that ED is one of the reasons for which a man will be under pressure wherein repeating unsuccessful attempts lead to psychological stress and a defective cycle (23), and with regard to limited literature on frequency of such a problem in men having type II diabetes in Iran and particularly in Mazandaran Province, the present study was to investigate the frequency of ED and its relationship with demographic and clinical variables.

## Methods

This study was a descriptive cross-sectional research on a population of married men aged between 20 and 70 years, suffering from type II diabetes diagnosed by an endocrinologist, and referring to the endocrinology clinics in the city of Sari. The sample size was estimated based on a single proportion design. It should be noted that a study with a sample of 350 diabetic patients would have a test power of 80% to detect a difference of 5% (45-55%) at a significant level of 0.05. 

The actual sample size obtained for this study was equal to 350 men with type 2 diabetes. The samples were also selected using consecutive sampling technique in 2017. The exclusion criteria consisted of patients with uncontrolled or acute CVDs within the last three months (heart attack, unstable angina, open heart surgery, etc.), individuals undergoing chemotherapy, prostate surgery, pelvic surgeries, uncontrolled endocrine diseases (like prolactinoma, Cushing syndrome, pituitary adenoma, etc.), sexual dysfunction prior to diabetes, and those using antiandrogens, narcotics, and stimulants (including opium, heroin, methadone, and amphetamine), as well as patients with recent consumption of psychiatric drugs (antidepressants, antipsychotics, and anticholinergics).

The patients were the asked to complete three validated questionnaires, namely the IIEF-15 and a demographic characteristic questionnaire at the clinics when they were on a waiting list by internal medicine residents. Privacy and confidentiality were also assured. After obtaining written consent from the patients to enter the study, demographic characteristic information such as age, number of children, number of family members, place of residence, residence status, level of income, job status, duration of suffering from diabetes, type of diabetes treatment, experience of thyroid disease and high blood pressure, experience of kidney and urinary tract surgery, as well as experience of psychiatric diseases and sexual problems and their probable treatments were firstly recorded using an already-prepared checklist. Then, the patients were requested to fill out the demographic characteristic questionnaire and the IIEF-15 as one translated into Persian whose validity and reliability had been evaluated (24, 25). 

The answers to this questionnaire were evaluated based on a five-point Likert-type scale (score 5 for natural function and score zero for the worst function) and the questions covered the following five domains of sexual function. So, the higher the score, the better the sexual function. The highest acceptable score was also by 75, indicating the best sexual status in different areas. This questionnaire was by itself divided into five smaller sub-scales as follows:

1. Erectile function (EF) is a sub-scale consisting of 6 items containing questions 1, 2, 3, 4, 5, and 15 of the main questionnaire. The score obtained from this sub-scale is described as 0-10 (severe), 11-16 (moderate), 17-21 (moderate to mild), 22-25 (mild), and 26-30 (without disorder). In addition, scores equal to or below 21 are perceived as presence of ED in the final analysis.
2. Orgasmic function (OF) is a sub-scale with questions 9 and 10 of the questionnaire whose score is between 0 and 10 in which score 10 shows the best function.
3. Sexual desire (SD) is a sub-scale containing questions 11 and 12 of the questionnaire whose score is between 2 and 10 in which higher scores are indicative of better function.
4. Intercourse satisfaction (IS) is a sub-scale comprising questions 6, 7, and 8 of the main questionnaire whose score is between 0 and 15 in which higher scores denote better function.
5. Overall satisfaction (OS) is a sub-scale referring to questions 13 and 14 of the main questionnaire whose score is between 2 and 10 in which higher scores represent better function (24, 25). The IBM SPSS statistics software (Version 22) was also used for data analysis. The quantitative variables were shown through descriptive methods such as mean±standard deviation (SD) and the qualitative ones were presented in the form of frequency. The chi-square test (Fisher’s exact test as required) was further employed to compare the qualitative variables.
6. To compare the presence of statistical differences between both study groups, one-way analysis of variance (ANOVA) was utilized. Levene’s test was additionally employed in cases where there was equal variance in the groups, and robust Welch test and Brown-Forsythe test were utilized to ensure the results of one-way ANOVA in cases where group variance was not equal. Moreover, if required, Games-Howell and Hochberg follow-up tests were used. In the present study, a p-value was smaller than 0.05 i.e. the significance level.

## Results

The total number of patients recruited in this study was 350. The most frequent age range of the participants was between 50 and 59 containing 165 patients (47.14%). The age range of other patients in terms of incidence rates was 90 patients (25.7%) above 59 years old, 67 individuals (19.1%) between 40 and 49 years old, 27 patients (7.7%) between 30 and 39 years old, and one individual (0.3%) aged between 20 and 29. The average time period of suffering from diabetes among these patients was 5.75±3.65 years (median=5, between 1 and 23). With regard to level of education in patients, 199 (57%) individuals did not have a high school diploma, 15 (4.3%) patients were illiterate, 10 (1.4%) of them had a Bachelor’s degree, 5 (1.4%) individuals had Associate’s degree, and 3 (0.9%) patients had Master’s and PhD degrees. The patients’ IIEF scores also varied between 5 and 71. The IIEF mean score was also equal to 43.79±16.98 (median=44). 

**Figure 1:**
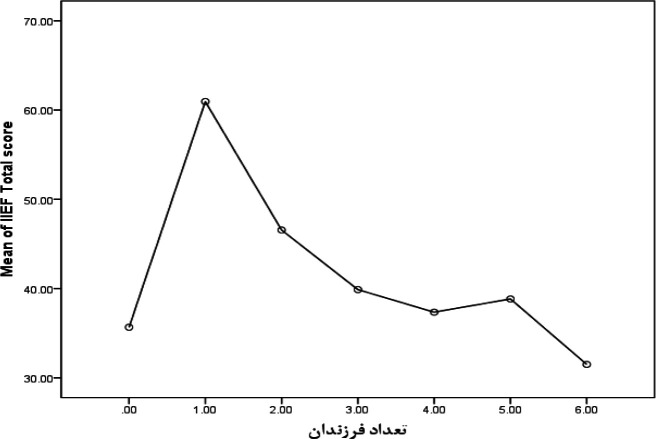
Changes in the IIEF mean scores based on age

The OF sub-scale was scored between 0 and 10. The mean score among the patients was also estimated to be 6.14±2.63 (median=6). The SD among the patients was 1.84±5.51 (median=6). The IS sub-scale was similarly scored between 0 and 15 whose mean was 2.91±7.44 (median=8) in this study. The score of OS was also between 2 and 10, and its mean in the present study was 2.58±6.58 (median=6). Regarding the investigation of EF sub-scale, 67 (19.20%) patients were without any dysfunctions, 64 (18.34%) individuals had mild dysfunctions, 80 (22.92) of them had mild-to-moderate dysfunctions, 100 (28.65%) patients had moderate dysfunctions, and 38 (10.89%) individuals were suffering from severe EF. Considering scores equal to or below 21, 218 (62.5%) patients were suffering from this dysfunction (table 1). As the patients’ age increased, the IIEF mean score also significantly decreased, indicating reduction of patients’ sexual function in older ages (p<0.001) (figure 1).

**Table 1:** Mean±SD of the IIEF and its sub-scales

**Sub-scales**	**Mean±SD**	**Median**	**Sub-scale ** **Score Range**
OF	6.14±2.63	6	0-10
SD	1.84±5.51	6	2-10
IS	2.91±7.44	8	0-15
OS	2.58±6.58	6	2-10
IIEF	43.79±16.98	44	0-75

The relationship between demographic and clinical factors and the IIEF mean score is illustrated in table 2. 

**Table 2:** Relationship between demographic and clinical factors and the IIEF total score

**P-value**	**IIEF total score** **(Mean±SD)**	**Factors **	
<0.001	70.00±0.0	20 to 29	Age(year)
	58.40±19.26	30 to 39	
	55.94±14.66	40 to 49	
	42.54±13.63	50 to 59	
	32.20±14.35	Above 59	
<0.001	33.93±20.97	Illiterate	Level of education
	41.14±15.63	Without high-school diploma	
	48.29±17.14	High-school Diploma	
	51.05±17.50	Associate’s degree and higher	
<0.001	35.66±29.93	No children	Number of children
	60.95±14.54	One child	
	46.55±16.50	2 children	
	39.87±15.00	3 children	
	37.35±17.25	4 children	
	38.83±17.54	5 children	
	31.50±33.23	6 children	
0.579	44.00±16.74	Urban	Place of residence
	42.56±18.44	Rural	
<0.001	47.86±16.64	Full-time	Job status
	48.60±20.04	Part-time	
	35.44±13.99	Retired	
0.038	45.17±16.30	Pills	Type of medication
	40.10±18.07	Insulin	
	39.21±19.56	Pills and insulin	
<0.001	48.59±16.89	Less than 5 years	Duration of suffering from diabetes
	39.23±14.55	Between 5 and 10 years	
	39.64±18.86	Between 10 and 15 years	
	44.00±22.56	More than 15 years	
0.021	44.87±16.84	Normal	Blood pressure
	39.88±16.96	High	
0.124	42.77±16.04	Natural	Body mass index (BMI)
	43.41±17.34	Overweight	
	50.26±15.18	Fat	
0.550	43.40±16.86	Normal	Cholesterol
	44.56±17.26	Impaired	
0.253	42.41±17.25	Normal	Triglyceride
	44.57±16.80	Impaired	
0.679	44.20±16.78	Normal	Fasting blood sugar (FBS)
	43.44±17.17	Impaired	
0.996	43.79±17.47	Normal	Hemoglobin A1c (HbA1c)
	43.78±16.65	High	

**Figure 2:**
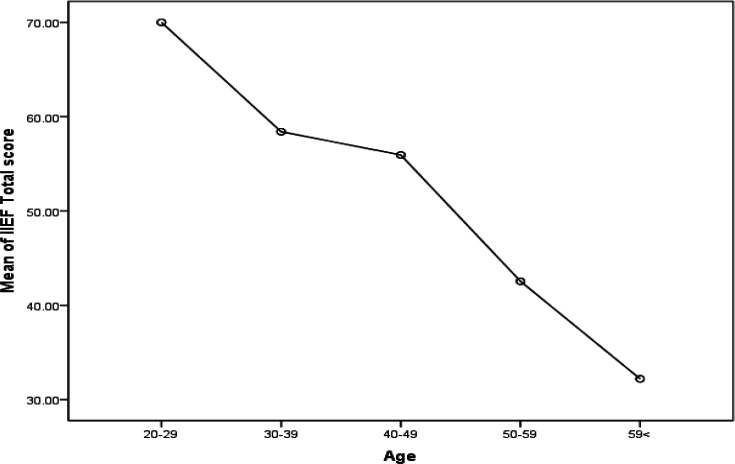
Changes in the IIEF mean scores based on number of children

As the patients’ level of education rose, the mean score of IIEF significantly increased, so it was concluded that sexual function and its satisfaction would boost as the level of education was added (p<0.001). The participants’ place of residence also had no effect on the IIEF mean score, and no statistically significant difference was observed between urban and rural areas (p=0.579). Regarding the relationship between number of children and sexual function, it was evident that individuals with one child and two children had better sexual function than others (p<0.001) (figure 2). 

Also, it was revealed that as the number of family members in this study increased, the IIEF mean scores of the patients enhanced as well, which was indicative of their better sexual function (p<0.001). Other factors affecting the quality of patients’ sexual function were level of income and job status, i.e. with an increase in level of income (p=0.005) and job security, the IIEF mean scores grew significantly (p<0.001). Having part-time or full-time jobs was also accompanied by better sexual function, and retirement was significantly associated with lower SD (p<0.001). The diabetic patients’ type of medication was similarly connected with their sexual function. The patients using insulin also had a significantly lower IIEF mean score (p=0.0.8). Experience of suffering from high blood pressure had significantly reduced the IIEF mean score in these patients and those not suffering from high blood pressure reported better sexual function (p=0.023). 

**Relationship between Demographic and Clinical Findings and ED: **A total number of 196 (77.2%) patients aged above 50 and 22 (23.2%) patients under 50 years old were suffering from ED. The probability of being affected with ED among the diabetic patients aged above 50 was 11.21 times as many as individuals under 50 (odds ratio (OR): 11.21, 95% confidence interval (CI): 6.40-19.62). The frequency of ED incidence among patients without high school diploma (p=0.002) and retired individuals (p<0.001) was significantly greater than others. Furthermore, with a growth in number of children, ED prevalence rate increased in a significant manner (p<0.001). 

The mean duration of suffering from diabetes among patients with ED was 5.96±3.42 years (between 1 and 23 years with median=5) and it was 5.41±4.01 years (between 1 and 20 years with median=4) among individuals without ED, so there was no statistically significant difference between them (p=0.174). 

However, no relationship was observed between patients’ place of residence (p=0.964) and level of income (P=0.283) and frequency of ED incidence. Although ED frequency among the patients with high blood pressure was greater, this relationship was not significant (OR: 1.64, 95% CI: 0.89-30.03, p=0.106). Moreover, regarding the type of medication, no significant difference was evident despite greater frequency of ED in patients receiving insulin (p=0.298) (Table 3).

**Table 3:** Relationship between demographic and clinical findings and ED

**P-value**	**Patients without ED**	**Patients with ED**	**Study data**	
<0.001	73 (76.8%)	22 (23.2%)	Under 50	Age (year)
	58 (22.8%)	196 (77.2%)	50 and more	
0.174	5.41±4.01		5.96±3.42	Average duration of suffering from diabetes
0.002	5 (33.3%)	10 (66.7%)	Illiterate	Level of education
	58 (29.1%)	141 (70.9%)	Without high-school diploma	
	57 (49.1%)	59 (50.9%)	High-school diploma	
	10 (55.6%)	8 (44.4%)	Associate degree and above	
<0.001	1 (33.3%)	2 (66.7%)	0	Number of children
	18 (90%)	2 (10%)	1	
	69 (43.4%)	90 (56.6%)	2	
	33 (26.4%)	92 (73.6%)	3	
	8 (23.5%)	26 (76.5%)	4	
	1 (16.7%)	5 (83.3%)	5	
	1 (50%)	1 (50%)	6	
0.964	112 (37.6%)	186 (62.4%)	Urban	Place of residence
	19 (37.3%)	32 (62.7%)	Rural	
<0.001	106 (48.6%)	112 (51.4%)	Full-time	Job status
	9 (60%)	6 (40%)	Part-time	
	16 (13.8%)	100 (86.2%)	Retired	
0.298	102 (39.8%)	154 (60.2%)	Pills	Type of medication
	24 (32.4%)	50 (67.6%)	Insulin	
	5 (26.3%)	14 (73.7%)	Pills and insulin	
0.106	17 (28.35)	43 (71.7%)	Yes	High blood pressure
	114 (39.4%)	175 (60.6%)	No	

## Discussion

The present study investigated the frequency of ED in a total number of 350 men suffering from type II diabetes. The results revealed that ED was present in 62.5% of the participants, and its severe and moderate types were observed in 40% of the patients. It should be noted that SD is of importance in diabetic patients, but it is often a neglected condition in these individuals although it has been reported in a high percentage of men and women with diabetes in recent studies (26, 29). In the study by Ziaei et al. (2010) in Iran on 200 patients (100 women and 100 men) suffering from type I and type II diabetes, prevalence rate of SD had been considerably high among them. In that study, 82.5% of the patients of both genders had experienced at least one type of SD (28). In 2007, Selvin had reported that ED in diabetic men in the United States had an incidence rate of over 50% (30). Another study in Netherlands had also demonstrated that the prevalence rate of SD among patients suffering from type II diabetes was about 41.3% (31). 

The studies conducted on Saudi diabetic patients had further concluded that moderate and severe types of ED had been observed in about 80-90% of patients (32, 33). According to an investigation by Malavige, 73.1% of men with diabetes had been affected with a degree of ED, while 84 (32.3%) individuals had been suffering from severe ED (34). Based on the study by Mofid et al. on 700 diabetic patients, 246 (35.1%) individuals had experienced ED (26). Besides, Walle et al. investigating the incidence rate of ED in 411 men with diabetes had reported it by 85.5% (35). The meta-analysis by Kouidrat et al. with the aim of reflecting on prevalence rate of ED in diabetic patients had correspondingly showed that the total prevalence rate of ED in patients with type I diabetes was 37.5%, it was 66.3% in patients with type II diabetes, and it was equal to 57.7% in all patients, which were similar to the results of the present study. 

Their investigation had demonstrated that the prevalence of ED using Sexual Health Inventory (SHI) was higher in men. In comparison with the healthy control population, ED had been very common among diabetic patients and involved more than half of the men with diabetes, and the probability of being affected with this condition among the diabetic population was 3.5 times more than healthy people (27). All in all, the prevalence rate of ED among men with diabetes varied between 35 and 90 (36). The high prevalence rate in the present study could be because of the fact that psychological factors affecting sexual function of diabetic patients had not been controlled. Regarding the high prevalence rate of depression in diabetic patients, removing such patients would result in the reduction of frequency and produce different results. 

Most of the patients in this study aged over 53 years, and on average, 5 years had passed since they had started experiencing diabetes. Most of them had a high school diploma or lower. A majority of them had no or one child and had also lived in families with 1 to 4 members. More than 32% of the patients had earned more than one million tomans per month, and most of them had been taking edible medications i.e. pills. 

The frequency of ED incidence was also correlated with lower level of education, retirement, and increase in age. The significant growth in the IIEF mean score was also correlated with lower age, increase in level of education, having one or two children, higher level of income, and having a job. On the other hand, the use of insulin, increase in age, experience of blood pressure, and having high blood pressure had significantly decreased the IIEF mean score. Besides ED and the IIEF mean score were not significantly correlated with BMI, cholesterol, FBS, thyroid stimulating hormone (TSH), thyroglobulin (Tg), and HbA1C. Based on the study by Malavige on 253 men suffering from type II diabetes, ED had been associated with lower level of income, older age, and duration of suffering from diabetes. Moreover, ED could be correlated with low level of education and high blood pressure (34). 

In Mofid’s research, the prevalence rate of ED had also increased from 9.7% at the age 20 to 30 to 43.3% in patients aged over 60. Consequently, in their investigation, increase in age had led to higher rates of ED, which was in line with the results of the present study. However, rising trend in duration of suffering from diabetes had augmented ED incidence in their study (26), whereas in the present study, the average duration of facing diabetes among patients with and without ED had revealed no significant difference. Other research studies had further demonstrated that age was a predictive factor for the prevalence of SD among diabetic patients. In his study, El-Sakka had also reported that 32% of men with diabetes aged under 50 and 67.6% of those over 50 had been suffering from ED (33). 

The effect of increase in age on the prevalence rate of SD among diabetic patients had been also demonstrated well in other studies (36, 37), denoting that chronic complications of diabetes such as hypertension could be related to growing incidence of SD among men and women with diabetes (38, 39). One of the limitations of the present study was that it was carried out with screening questionnaires rather than through psychiatric clinical interviews. Therefore, it was recommended to design and conduct a study on two groups including diabetic patients and healthy people as a control group homogenized in terms of demographic characteristics and socio-economic conditions in the future, for a more accurate investigation of the effect of diabetes on sexual function. Furthermore, levels of testosterone were not measured in this study. Subsequent studies concentrating on all factors affecting SD in diabetic patients are thus recommended to do so. Consequently, later studies in this field should consider all mentioned limitations, and it is suggested to develop prospective and longitudinal research in the future. 

In conclusion according to the finding of the present study, the prevalence rate of SD in men suffering from type II diabetes was high, and controlling blood sugar was not correlated with the frequency of SD in the population under study. These findings suggested that SD must be carefully examined in healthcare clinics for diabetic patients. It is also required to conduct ED screening for such patients, especially elderly ones experiencing diabetes, so that ED can be diagnosed and treated. Moreover, raising patient awareness as the first and the most effective way for prevention and treatment is of utmost importance.

## Conﬂict of Interest:

The authors of this article have no conflicts of interest.
